# Real-time detection of dielectric anisotropy or isotropy in unconventional oil-gas reservoir rocks supported by the oblique-incidence reflectivity difference technique

**DOI:** 10.1038/srep39306

**Published:** 2016-12-15

**Authors:** Honglei Zhan, Jin Wang, Kun Zhao, Huibin Lű, Kuijuan Jin, Liping He, Guozhen Yang, Lizhi Xiao

**Affiliations:** 1 State Key Laboratory of Petroleum Resources and Prospecting, China University of Petroleum, Beijing, 102249, China; 2 Beijing Key Laboratory of Optical Detection Technology for Oil and Gas, China University of Petroleum, Beijing, 102249, China; 3 Institute of Physics, Chinese Academy of Sciences, Beijing, 100190, China

## Abstract

Current geological extraction theory and techniques are very limited to adequately characterize the unconventional oil-gas reservoirs because of the considerable complexity of the geological structures. Optical measurement has the advantages of non-interference with the earth magnetic fields, and is often useful in detecting various physical properties. One key parameter that can be detected using optical methods is the dielectric permittivity, which reflects the mineral and organic properties. Here we reported an oblique-incidence reflectivity difference (OIRD) technique that is sensitive to the dielectric and surface properties and can be applied to characterization of reservoir rocks, such as shale and sandstone core samples extracted from subsurface. The layered distribution of the dielectric properties in shales and the uniform distribution in sandstones are clearly identified using the OIRD signals. In shales, the micro-cracks and particle orientation result in directional changes of the dielectric and surface properties, and thus, the isotropy and anisotropy of the rock can be characterized by OIRD. As the dielectric and surface properties are closely related to the hydrocarbon-bearing features in oil-gas reservoirs, we believe that the precise measurement carried with OIRD can help in improving the recovery efficiency in well-drilling process.

Oil-gas industry has been shifting exploration and development activities from conventional hydrocarbon accumulations to unconventional ones. The research of unconventional oil-gas resources focus on the reservoir space and whether the trap contains petroleum. The physical and structural properties of dense reservoir rocks are related to the recovery efficiency of oil-gas distribution in well-drilling process. Black- or dark-shale deposits in the foreland reflect distinct peculiarities of the deformational activity in any one Paleozoic orogeny because of rapid, loading-related, lithospheric subsidence and sediment starvation in the resulting basins^1^,^2^. The dielectric property of the rock is an important reference for geoscientists and petrophysicists to more precisely and effectively evaluate oil-gas reservoirs before and during exploration. Because of the quiet and deep anaerobic depositional environment that developed over thousands of years, shale often exhibits well-developed layered structures. The laminations in the layered structures are nearly parallel to each other. The significance of these laminations can be revealed by the geo-mechanics research and extraction of unconventional resources^3^. Generally, the mechanical properties are weakest at the laminations, which can withstand relatively small shear stresses. As a result, the stability of the well wall is degraded^4^. Moreover, porosity and fractures exist near the bedding planes. These unique reservoir structures indicate the existence characteristics of the oil-gas molecules, with significant variations in terms of the permeability in different directions. In sonic logging, the propagation of acoustic waves reflects the obvious anisotropy in the geological strata^5^. After obtaining and precisely analyzing these logging data, hydraulic fracturing technology can be employed. In this technique, fracturing fluid is poured into the reservoir at a pressure that exceeds the absorbing capacity of the oil reservoir. Regarding the location at which the fracturing fluid is poured, the micro-cracks perpendicular to the laminations can be selected to obtain more and larger cracks, thereby creating more fractured channels for hydrocarbon molecules to be extracted. Shale’s layered structure is highly significant for traditional mining technologies^6^. Sandstone represents another significant component of tight gas reservoirs, with one-fourth of the world’s sedimentary rocks estimated to be sandstone, which mainly consists of sand-sized particles. Sandstone also has a low absolute permeability (between ~ 10 μD and 0.5 mD) and a connected porosity less than 10% because of its tight structure. In contrast to shale, sandstone rarely possesses an obvious layered structure. Regarding the research on sandstone reservoirs, the development and preservation of the pores has been the central topic relating to oil-gas reservoirs and are practical problems that must be solved in sedimentary basins^7^,^8^,^9^.

The characterization of rock anisotropy is significant for hydrocarbon exploration. Electron microscopy techniques are commonly used for microstructure determination. The planes perpendicular and parallel to the bedding can be analyzed by scanning electron microscopy (SEM), and SEM images have shown some patchy kaolinite and acicular illite with preferred orientations parallel to the bedding^10^,^11^. High-resolution fractures can also be identified by SEM imaging analysis. Micro-computed tomography (micro-CT) is also an effective method to characterize pore and throat structures in a three-dimensional space. By combining CT scanning and digital image processing technology, the meso-damage characteristics of rock can be investigated during the process of shale hydration^12^. However, such CT-based approaches require sample pretreatment.

We employed an optical technique, referred as oblique-incidence reflectivity difference (OIRD), of imaging the shale and sandstone surfaces for improved characterization of the anisotropy of shale and the isotropy of sandstone. In recent years, various optical techniques have emerged as important tools for evaluating the potential of reservoirs and for characterizing oil-gas and pollutant properties^13^,^14^. Representative newly developed techniques include terahertz (THz) spectroscopy^15^,^16^, all-optical detection^17^, and remote sensing^18^. Similar to the optical methods mentioned above, OIRD is insensitive to the interference of subsurface electromagnetic fields and to high subsurface temperature and pressure. Compared with these methods, the OIRD technique is a more sensitive detection tool. OIRD signal depends on the dielectric and surface properties of the rock surface because this technique measures the difference in reflectivity between *s-* and *p-*polarized lights. At present, OIRD is mainly employed in biological molecules identification and imaging. OIRD scan was employed to detection a microarray of 60-base oligonucleotides before and after hybridization. OIRD image can reveal the hybridization and determine the thickness of oligonucleotides in the microarray, simultaneously^19^. Based on a widely used OIRD analysis formula expressed as


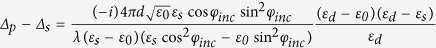


where *ε*_0_, ε_s_, *φ*_*inc*_ and *λ* represent the relative permittivity of environment, relative permittivity of basement, incidence angle and wavelength, thus OIRD signal intensity is a function of dielectric constant *ε*_*d*_ and effective thickness *d*^20^. OIRD has a time resolution of 20 microseconds, a spatial resolution of 0.4 nm, and a detection sensitivity of 14 fg of protein per spot, according to previous reports^21^,^22^. OIRD can be employed for the *in situ* and real-time detection of oxide film epitaxial growth^23^. Recently, more promising applications were reported for the label-free, high-throughput detection of the interactions and dynamic processes of biological molecules and for the identification of the defects as well as the adsorption in simulated reservoirs^24^,^25^. In this research, we applied the OIRD method to quantify the surface properties of subsurface shale and sandstone cores. Our results indicated that the anisotropy in shale and isotropy in sandstone can be clearly identified by OIRD. The experimental results suggest that the OIRD technique can be used for core analysis required during oil-gas exploration and development, especially in the unconventional petroleum resource industry.

## Results

**Figure 1:**
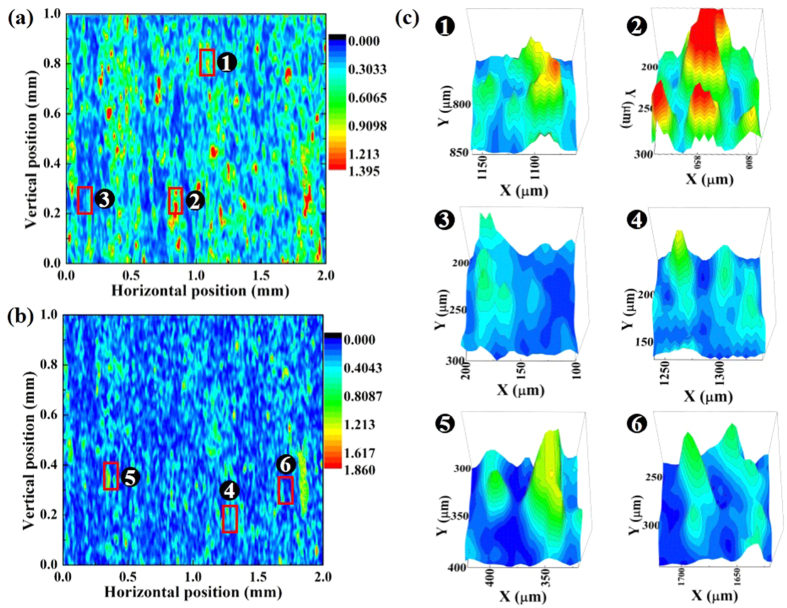
OIRD images of shale core when the scan direction is parallel to the bedding of shale. (**a**) 2-D image of Re{*Δ*_*p*_ − *Δ*_*s*_}. A number of striations are obtained parallel to the 1-mm side and perpendicular to the 2-mm side. (**b**) 2-D image of Im{*Δ*_*p*_ − *Δ*_*s*_}. A similar number of striations is obtained parallel to the 1-mm side and perpendicular to the 2-mm side but show different intensities compared with (**a**). (**c**) 3-D images of Re{*Δ*_*p*_ − *Δ*_*s*_} of six selected regions with areas of 100 μm × 100 μm. Within the six regions, three are from (**a**), and the others are from (**b**). The agreement between the OIRD signals and the bedding properties of the shale is validated.

Lamination, or a layered structure, is one of the most significant features of shale. The shale rocks from most areas have obvious layered structures. Because of the existence of the bedding surface, the mechanical properties of shale are markedly different from those of rocks with homogeneous structures. Generally, the weakness associated with cementation results in the damage to the lamination surface to precede the damage to the body. Moreover, weak planes cause instability of the wellbore during exploration. A rock core is typically used for to analyze the underground geology and mineral resources. In this research, a shale core with a layered structure was measured and analyzed using OIRD. Initially, the sample was located on a two-dimensional (2-D) stage, and we set the direction of sample movement parallel to the bedding plane and adjusted the phase shifter and polarization analyzer to make the *I(2* *Ω*) and *I(1* *Ω*) signals equal to zero. Next, the sample was 2-D scanned in a randomly selected region of 2 mm × 1 mm. During the scanning measurement, the real and imaginary signals, Im{*Δ*_*p*_ − *Δ*_*s*_} and Re{*Δ*_*p*_ − *Δ*_*s*_}, and their relative positions were recorded by a computer^23^ (Extended Data Figure 1). Figure 1a,b show the signal intensity images of Re{*Δ*_*p*_ − *Δ*_*s*_} and Im{*Δ*_*p*_ − *Δ*_*s*_}, respectively, obtained from a shale core. The signal intensity is different at different positions, revealing the position-dependent variance of the dielectric properties on the shale core surface. A number of striations were parallel to the 1-mm side and perpendicular to the 2-mm side. Close agreement can be observed between the real map and the imaginary images. The width of the adjacent main beddings was ~100–300 μm in both images. The secondary laminations were found to have a width ranging from several to dozens of microns. These beddings appeared to be nearly parallel to each other; in fact, some of them were discontinuous. To discuss the lithology more systematically, three dimensional (3-D) cross-sections of the OIRD Re{*Δ*_*p*_ − *Δ*_*s*_} of a selected area were extracted, and the sections with high intensities were observed to be perpendicular and parallel to the bedding directions (Extended Data Figure 2). By connecting the peaks, we can obtain the main bedding plane of shale anisotropy that is perpendicular to the X-axis with an interval of ~300 μm. Figure 1c shows the 3-D images of six randomly selected regions in the real and imaginary images of the shale sample. The area of all six selected regions equaled 100 μm × 100 μm, and two or three beddings can be clearly observed in these figures. In all six areas, the widths of the peak areas and the distance between the consecutive peaks were 5–30 μm, in agreement with the actual layered structures of shale.

**Figure 2:**
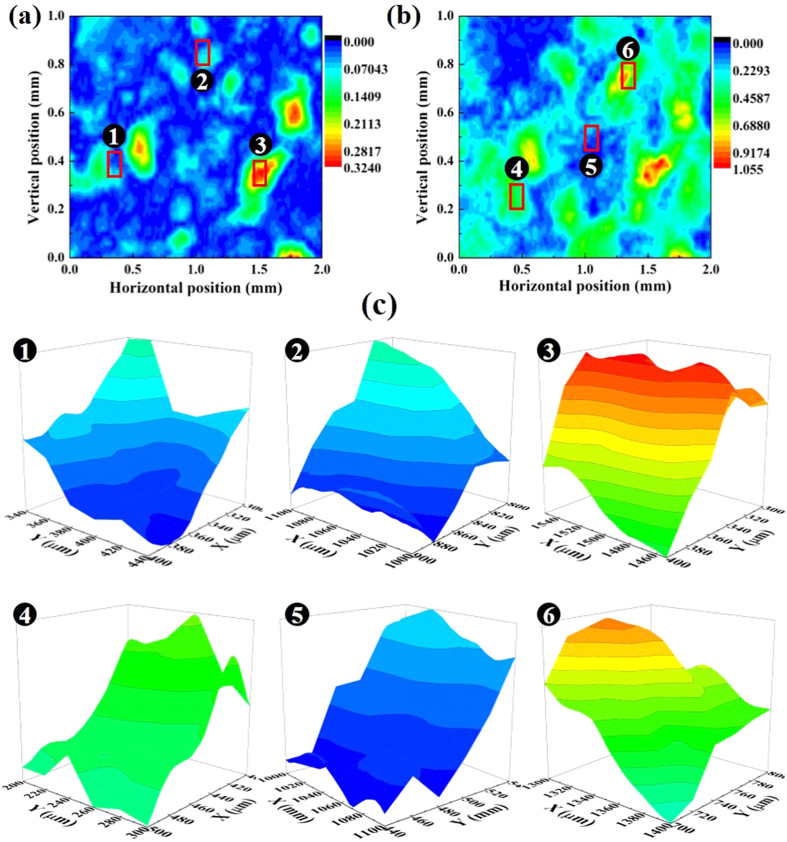
OIRD images of a sandstone core in which a random region was scanned by OIRD. (**a**) 2-D image of Re{*Δ*_*p*_ − *Δ*_*s*_} of sandstone. No striation was observed. In most areas, the signal intensities were uniform; in addition, several peak points existed, but they were not connected. (**b**) 2-D image of Im{*Δ*_*p*_ − *Δ*_*s*_} of sandstone. No striation was observed. In most areas, the signal intensities were uniform; in addition, several peak points existed, but they were not connected. (**c**) 3-D images of Re{*Δ*_*p*_ − *Δ*_*s*_} and Im{*Δ*_*p*_ − *Δ*_*s*_} of six selected regions with areas of 100 μm × 100 μm. Areas No. 1, 2 and 3 are shown in (**a**), and No. 4, 5 and 6 are shown in (**b**). The information gathered by this technique indicated the surface properties of the sandstone.

Similarly, sandstone, another dense rock that has been widely studied by many geoscientists, is investigated in this paper. Sandstone is a sedimentary rock and is mainly composed of sand grains. Through a series of processes, such as weathering, erosion and transportation, sandstone gradually formed in basins. In contrast to shale, sandstone rarely has a clear layered structure. Because the composition of sandstone is relatively homogeneous, it is generally considered to be isotropic. Similar to the process for shale, we obtained the real and imaginary OIRD signals in selected areas of the sandstone (Extended Data Figure 3). The signal intensities exhibited several peaks on the surfaces in both the real and imaginary images, showing the position-dependent uniformity of the physical properties. Figure 2a,b depict the signal intensity images of Re{*Δ*_*p*_ − *Δ*_*s*_} and Im{*Δ*_*p*_ − *Δ*_*s*_} obtained from the sandstone core. In contrast to the shale images presented in Fig. 1, sandstone core does not exhibit any lamination, confirming that the measured striations in Fig. 1 are entirely caused by shale’s layered structure. It is apparent that sand grains with different sizes are randomly distributed throughout the surface of sandstone. Large granules can have widths as large as ~400 μm size. In addition, the locations of the grains in the real image are reflected in the imaginary image. We also plotted the enlarged OIRD signals of six randomly selected regions with areas equaling 100 μm × 100 μm. In the selected areas, the signals continuously changed at near positions. Therefore, OIRD shows great consistency and stability when used to characterize rock surfaces and dielectric properties.

## Discussion

**Figure 3:**
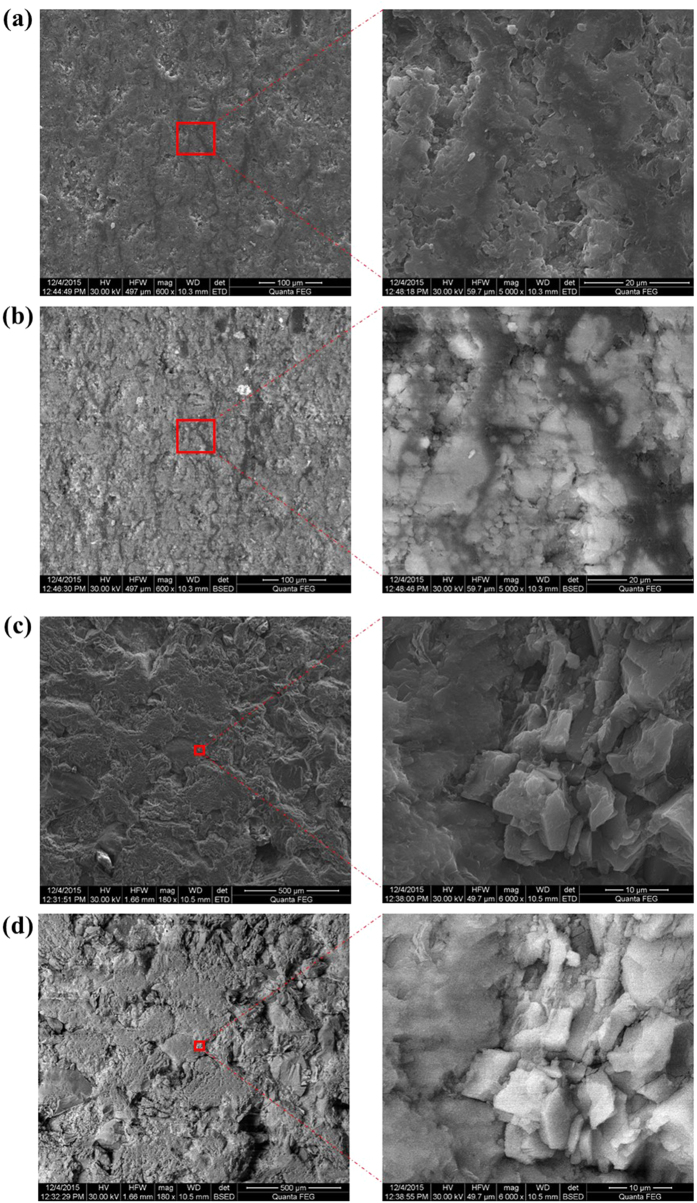
SEM images of shale and sandstone samples that were measured by OIRD. (**a**) SE morphology contrast images of shale: the right image shows an enlarged view of the selected area in the left image. A directed crevice can be observed in the right image. (**b**) BSE images of shale: the right image is an enlarged view of the selected area in the left image. An orientational arrangement of the same particles (black) can be observed parallel to the Y-axis. (**c**) SE morphology contrast images of sandstone: the right image shows an enlarged view of the selected area in the left image. A directed crevice is observed in the right image. An annular crack that is different from the directed crevice in shale is observed. (**d**) BSE images of sandstone: the right image is an enlarged view of the selected area in the left image. A symmetrical arrangement of the same particles (white) can be seen in this image.

The layered structures of shale and the approximate isotropy of sandstone are clearly reflected in Figs 1 and 2. The rocks exhibit directionality in the OIRD signal intensities. The anisotropy of the rock can be divided into two types: one type is based on the existence of micro-cracks and their arrangements in different directions and is referred to as stress anisotropy; the other type relates to the orientation of the rock particles and does not change with stress. Herein, the phenomena of the layered structures of shale and the approximate isotropy of sandstone were directly validated by optical microscopy (Extended Data Figure 4). To detect the componential and structural information of the rock surfaces, SEM was then employed to analyze the shale and sandstone cores investigated with OIRD. Secondary electron (SE) and back-scattered electron (BSE) modes were used to obtain new set of images. Figure 3 shows the SEM images of shale and sandstone cores at several different scales: (a) and (c) show the SE morphology contrast images of shale and sandstone, respectively, with relatively large and small sizes; (b) and (d) present the BSE images of shale and sandstone with different scales. SE was very sensitive to the surface morphology, thus making it an effective method for observing the surface morphology. The contrast of BSE imaging is caused by the different atomic masses. In the first image in Extended Data Figure 5, a number of stripes can be observed and are parallel to each other. The other images represent magnifications of the stripes. In Fig. 3, a series of micro-cracks can be clearly distinguished in grayscale; the right image shows an approximately straight crack with a width of 5–10 μm that was related to the OIRD peaks shown in the Fig. 1. The BSE map reflects the distribution of components with the same atomic mass. As shown in Fig. 3b, oriented lines shown in the same color are parallel to each other; thus, the black areas confirm the arrangement of the orientation of one component with a relatively small atomic mass. The orientation of a higher-atomic-mass component is shown in white (Extended Data Figure 6). The SE and BSE images validated the micro-cracks and orientations of the rock particles in the shale, as clearly shown in the OIRD images with the corresponding real and imaginary signals. We also performed SEM on the surface of sandstone and enlarged the area gradually, as shown in Extended Data Figure 7. In Fig. 3c, the SE imaging shows some differently sized particles with clear annular cracks between them. The shapes of cracks in sandstone were different from those in shale. As a whole, sandstone can be considered as isotropic because of the absence of striations and lack of orientation in the particle arrangement. It can be observed in Fig. 3d that the components with the same or similar atomic masses are uniformly distributed throughout sandstone. The white areas correspond to particles with larger atomic masses, which are randomly arranged and not oriented. SEM imaging based on SE and BSE validated the distribution characteristics of the real and imaginary signals of OIRD. Thus, this technique is a very promising and practical technology to detect isotropy and anisotropy in rock and is a convenient supplementary method that can be combined with conventional methods.

This study focused on applying OIRD to shale and sandstone rocks. We clearly observed the anisotropy of shale and isotropy of sandstone in the images of the Re{*Δ*_*p*_ − *Δ*_*s*_} and Im{*Δ*_*p*_ − *Δ*_*s*_} signal intensities. In shale, the width of the adjacent main beddings was ~100–300 μm; in addition, the secondary laminations had widths ranging from ~30 to ~50 micron. The detection results in the OIRD images are in agreement with those of the optical microscopy images (Extended Data Figure 4) and SEM maps (Fig. 3 and Extended Data Figures 5–7). Accordingly, this study suggests that the methods used to characterize the isotropy or anisotropy of reservoir rock should combine previous techniques, such as sonic wave, and newly developed optical methods, such as OIRD, which reflects the dielectric properties and distributions of rock. Undoubtedly, as exploration continues to deepen, the production of conventional oil and natural gas resources will become increasingly difficult; thus, characterization of rock is becoming important. There is no reason to keep unconventional oil and gas resources ‘sleeping’. The anisotropy in shale and isotropy in sandstone strongly influence various aspects of exploration, such as drilling efficiency and borehole deviation. For example, because of the different strength properties of the stratification layers, the workload of the rock layer increases during drilling; thus, the anisotropy of the rock will influence the drilling efficiency. In addition, because of the anisotropy of the rock, the drill pipe will become mechanically unbalanced, and as a result, the drill rod may deflect at a certain angle and even bend, thereby affecting the deviation of the drilling hole. During oil-gas extraction, the distribution of the strata or rocks’ mechanics is the primary feature that should be characterized. Near cracks, the effective density and thickness are clearly different from those at other locations. In the micro-structure, from rock to cracks and from cracks to rock, the dielectric and surface properties undergo repeated variations. As reported in previous articles, the OIRD signal, which reflects the difference between the reflectivity of *s-* and *p-*polarized light, is closely related to the sample’s dielectric and surface properties, and as a result, OIRD is a very sensitive surface detection technique. OIRD is a very promising and practical technology for detecting the isotropy and anisotropy in rock and is a convenient supplementary technique for conventional methods.

## Methods

Similar to some other technologies, there also exist some limitations of the new technique. For example, the baseline of signal should be regulated in each measurement; more mathematical methods should be developed to analyze the data of signal. Overall, the research proves that OIRD is a very promising and practical technology for detecting the isotropy and anisotropy in rock so that it can be used for core analysis during oil-gas exploration. Extended Data Figure 8 shows a schematic of the OIRD experimental setup for the detection of rock, including shale and sandstone. The probe beam from a He-Ne laser with wavelength of λ = 632.8 nm is initially *p* polarized. After reflection by two mirrors (M1 and M2), the laser passes through a polarizer (P) to ensure p-polarized incidence. A photoelastic modulator (PEM) is then employed to oscillate the polarization of the laser beam between *p-* and *s-*polarization at a frequency of 50 kHz. The laser beam output from the PEM enters a phase shifter (PS), which is utilized to create a fixed phase difference *Φ*_*ps*_between the *p-* and *s-*polarization components. After focusing by an optical lens (L1), the laser reaches the surface of the rock to be measured at a incidence angle of *θ* = 60°. The reflected beam passes through a second lens (L2) that collects the diffuse light into a parallel beam. The light is then introduced into a polarization analyzer (PA) with the transmission axis set at *θ*_*A*_ from *s-*polarization. The intensity of the reflected beam is detected by a silicon photodiode (PD). Two lock-in amplifiers are used to measure the first harmonic *I(1Ω*) and second harmonic *I(2Ω*) signals. Before the scanning measurement, we adjust the phase shifter and polarization analyzer so that the *I(2Ω*) and *I(1Ω*) signals are equal to zero to improve the sensitivity of the measurement. During the scanning measurement, the *I(2Ω*) and *I(1Ω*) signals of Im{*Δ*_*p*_ − *Δ*_*s*_} and Re{*Δ*_*p*_ − *Δ*_*s*_} are recorded by a computer. The *I(2Ω*) and *I(1*Ω) signals include information about the surface and the dielectric properties of the sample^24^,^26^.

Generally, there are no special requirements on samples preparation for OIRD detection. To obtain the OIRD images of the rock surface, a 2-D motorized stage is employed. The rock is fastened on the upper surface of the stage, which is controlled by special software. The interval between two adjacent scans can be 1 μm. In the experiment, the scanning step length was 4 μm. As shown in Extended Data Figure 9, the scan directions of the laser or the movement directions of the rocks were specifically chosen. In this research, shale and sandstone cores were used. Each core is a cylinder of 25-mm diameter and 50-mm long. Shale was cored at a depth of 3325.4 m in the horizontal direction; thus, the petrophysical layers are perpendicular to the upper and lower surfaces of the core. Sandstone is the main reservoir of oil, gas and groundwater. The shale and sandstone cores were cut and then polished to confirm the parallelism between the upper and lower surfaces of the cores before OIRD measurement. Shale has a vectored stratification plane over the whole space. The direction was confirmed to be parallel relative to the bedding planes during the scan. For the sandstone, however, obvious vectored bedding planes do not exist in 3-D space, and thus, the area to be measured was randomly selected on the surface. All measured regions were rectangular planes of 2 mm × 1 mm. The measurements were performed under ambient conditions.

## Additional Information

**How to cite this article**: Zhan, H. *et al*. Real-time detection of dielectric anisotropy or isotropy in unconventional oil-gas reservoir rocks supported by the oblique-incidence reflectivity difference technique. *Sci. Rep.* **6**, 39306; doi: 10.1038/srep39306 (2016).

**Publisher’s note:** Springer Nature remains neutral with regard to jurisdictional claims in published maps and institutional affiliations.

## Supplementary Material

Supplementary Information
