# Supplementary material for: Real-time detection of dielectric anisotropy or isotropy in unconventional oil-gas reservoir rocks supported by the oblique-incidence reflectivity difference technique
Source: Sci Rep. 2016 Dec 15;6:39306. doi: 10.1038/srep39306 (PMC5157029; doi:10.1038/srep39306)
Supplement: Supplementary Information [file srep39306-s1.doc]

**Supplementary Information for**

**Real-time detection of dielectric anisotropy or isotropy in unconventional oil-gas reservoir rocks supported by the oblique-incidence reflectivity difference technique**

Honglei Zhan^1,2^, Jin Wang^2^, Kun Zhao^1,2,*^, Huibin Lű^3,*^, Kuijuan Jin^3^, Liping He^3^, Guozhen Yang^3^ and Lizhi Xiao^1^

*^1^State Key Laboratory of Petroleum Resources and Prospecting, China University of Petroleum, Beijing 102249, China;*

*^2^Beijing Key Laboratory of Optical Detection Technology for Oil and Gas, China University of Petroleum, Beijing 102249, China;*

*^3^Institute of Physics, Chinese Academy of Sciences, Beijing 100190, China*

Corresponding authors: email address: [zhk@cup.edu.cn](mailto:zhk@cup.edu.cn), Tel.: +86 010 89732270;

email address: [hblu@aphy.iphy.ac.cn](mailto:hblu@aphy.iphy.ac.cn)


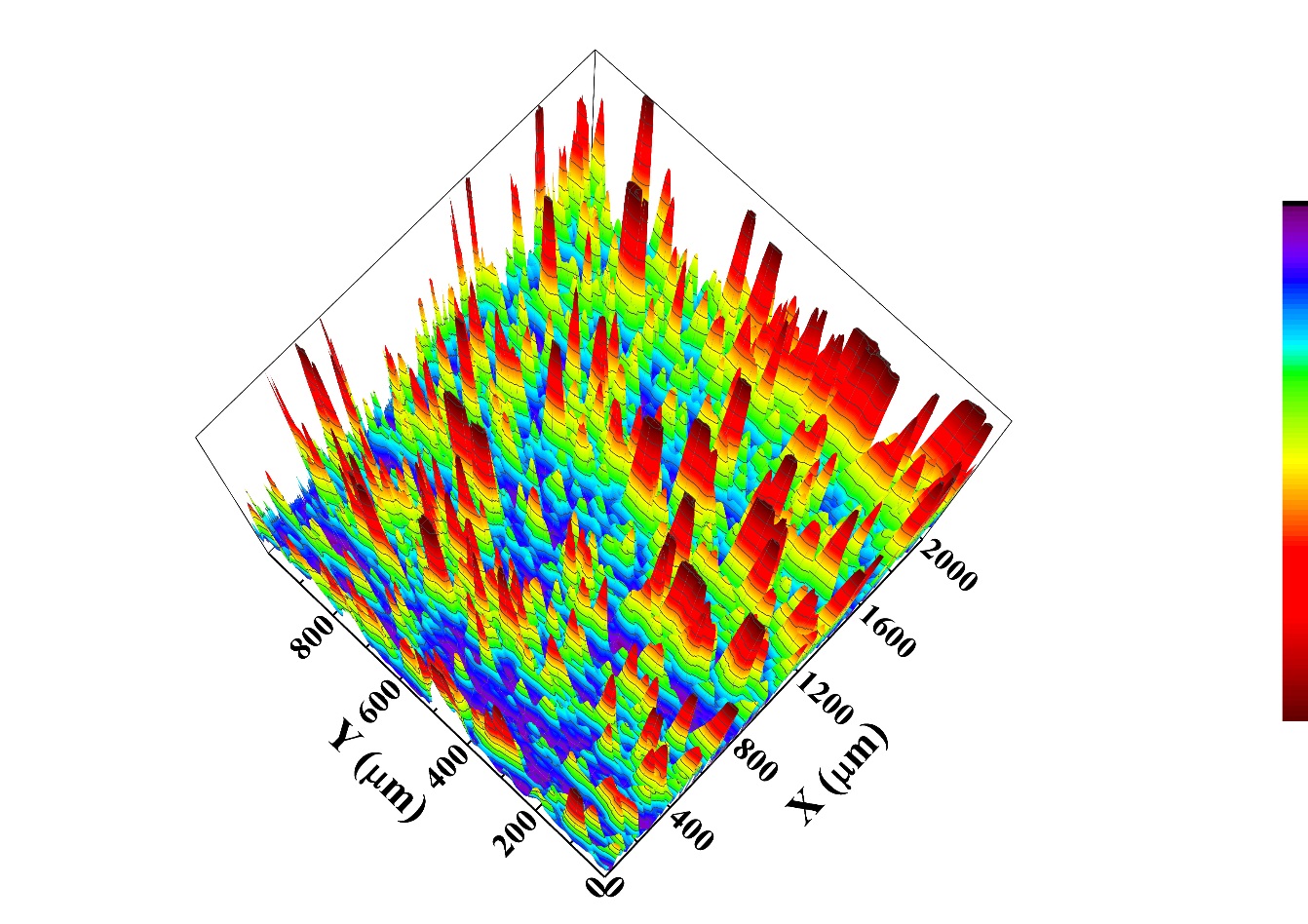


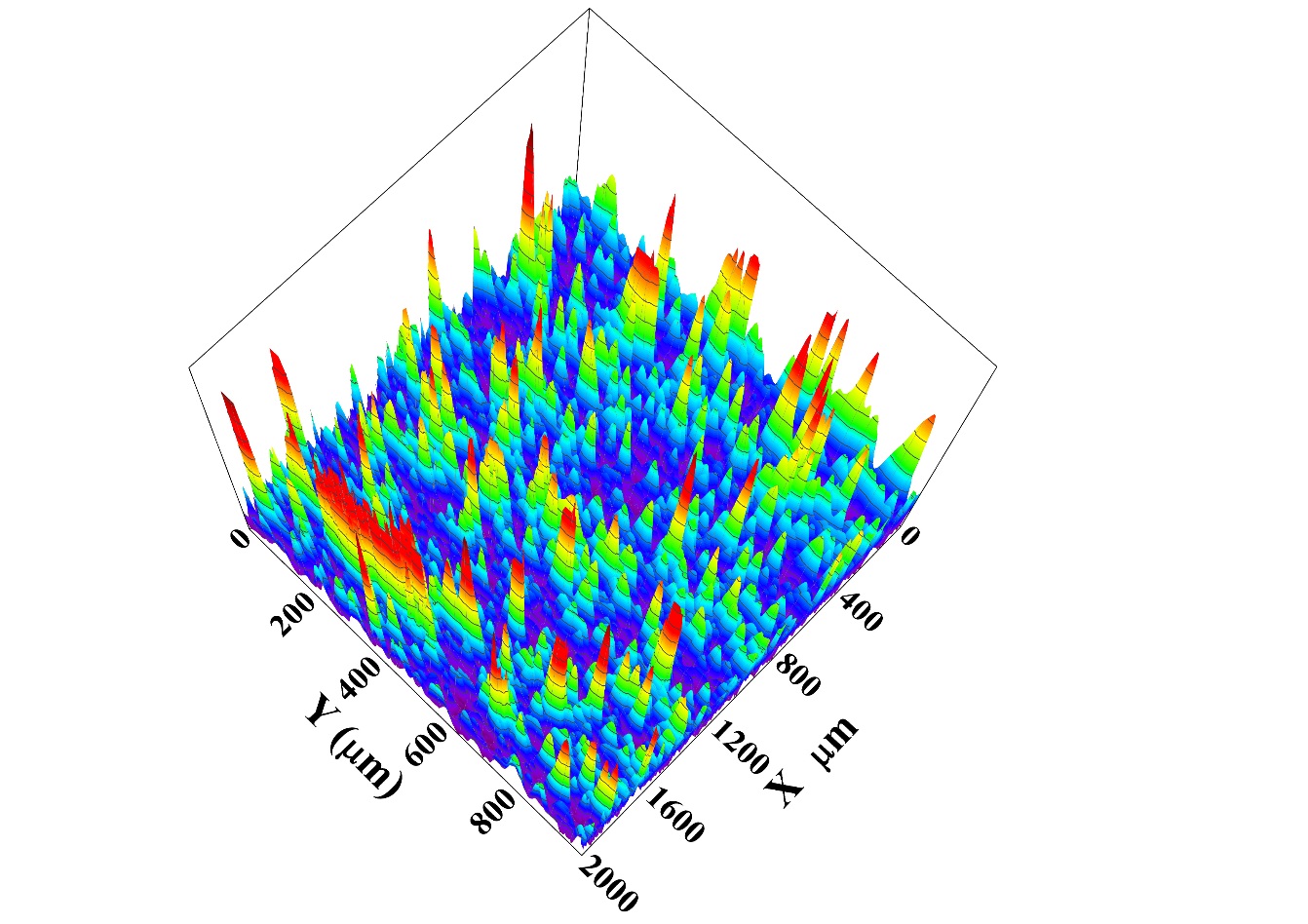


**Extended Data Figure 1. Position-dependent OIRD signals of shale.** Real intensities at different scanning positions (up), where the Re*{Δ_p_-Δ_s_}* values reflect the relative variance. Shale’s imaginary intensities at different scanning positions (down), where the Im*{Δ_p_-Δ_s_}* values reflect the relative variance. In the 3-D systems of both the real and imaginary parts, the same bedding information can be observed. These beddings appear to be approximately, but not absolutely, parallel to each other; in fact, some are discontinuous. Generally, the beddings are parallel to the 1-mm sides. Moreover, the bedding lines are successive in some areas but not discontinuous in others, reflecting the complex composition and structure on the shale surface.


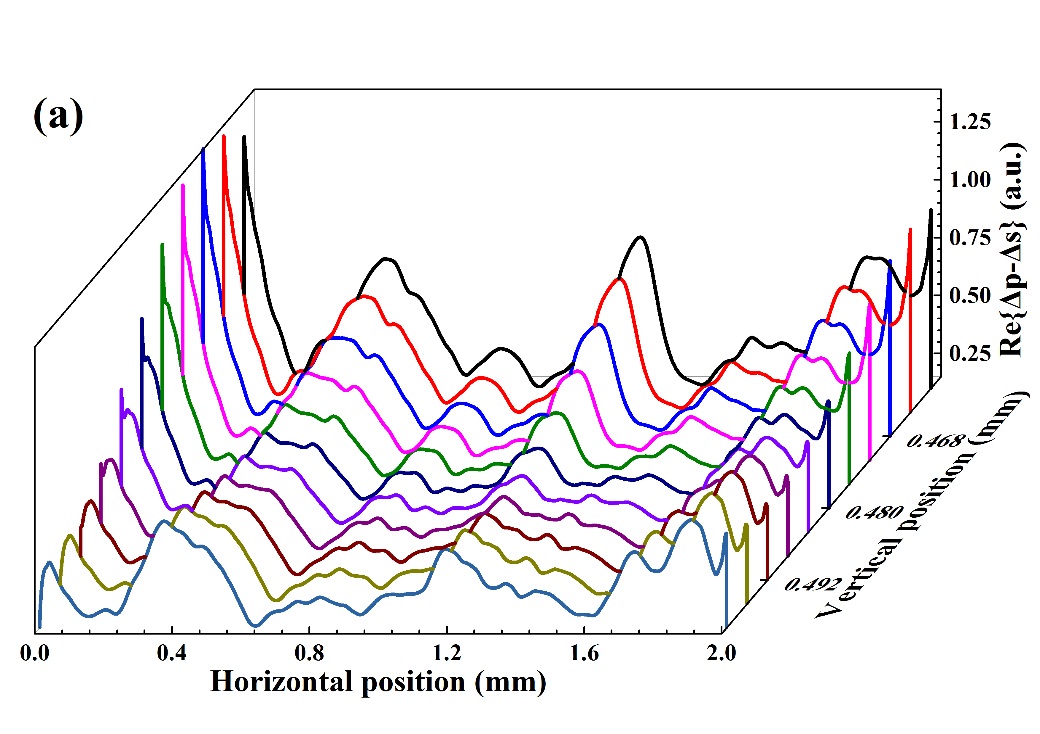

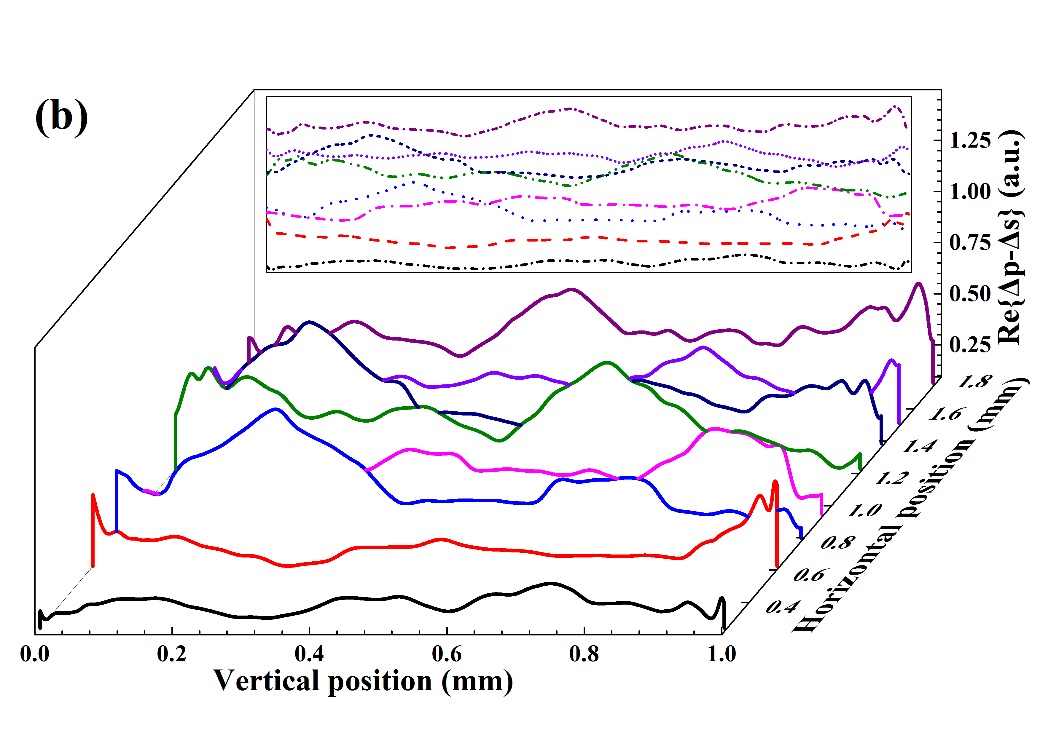


**Extended Data Figure 2. Cross-sectional analysis of the OIRD Re*{Δ_p_-Δ_s_}* signals of shale.**(a) Cross section of the horizontal plane of the OIRD real signals. (b) Cross section of the vertical plane of the OIRD real signals of shale. Shale exhibited obvious anisotropy between the horizontal (X-axis) and vertical (Y-axis) directions. This anisotropy occurs perpendicular and parallel to the bedding directions. To more intuitively observe the layered structure and achieve a higher signal-to-noise ratio, the OIRD data were pre-processed using the Adjacent-Averaging filter. This data pretreatment reduces the noise and smoothes the curves but does not distort the waveforms and features. One of the most important features in Extended Data Figure 2(a) is the Re{*Δp*-*Δs*} peaks, which are located at the same vertical positions. The unique horizontal positions of these peaks were ~ 0.01, 0.41, 0.76, 1.16, 1.48, and 1.8 mm, respectively. Intervals of ~300 µm are obtained. Connecting the peaks, we can determine the main bedding plane of the shale anisotropy, which is perpendicular to the X-axis. However, the OIRD signals of the cross section parallel to bedding planes in Extended Data Figure 2(b) indicated small difference relative to that in Fig. 5(a) over the vertical position range. The largest value was ~ 0.5 mm, and the average value over the whole range was approximately 0.25 mm. These results indicated that the approximate isotropic properties of the cross sections are parallel to shale’s laminations.

**
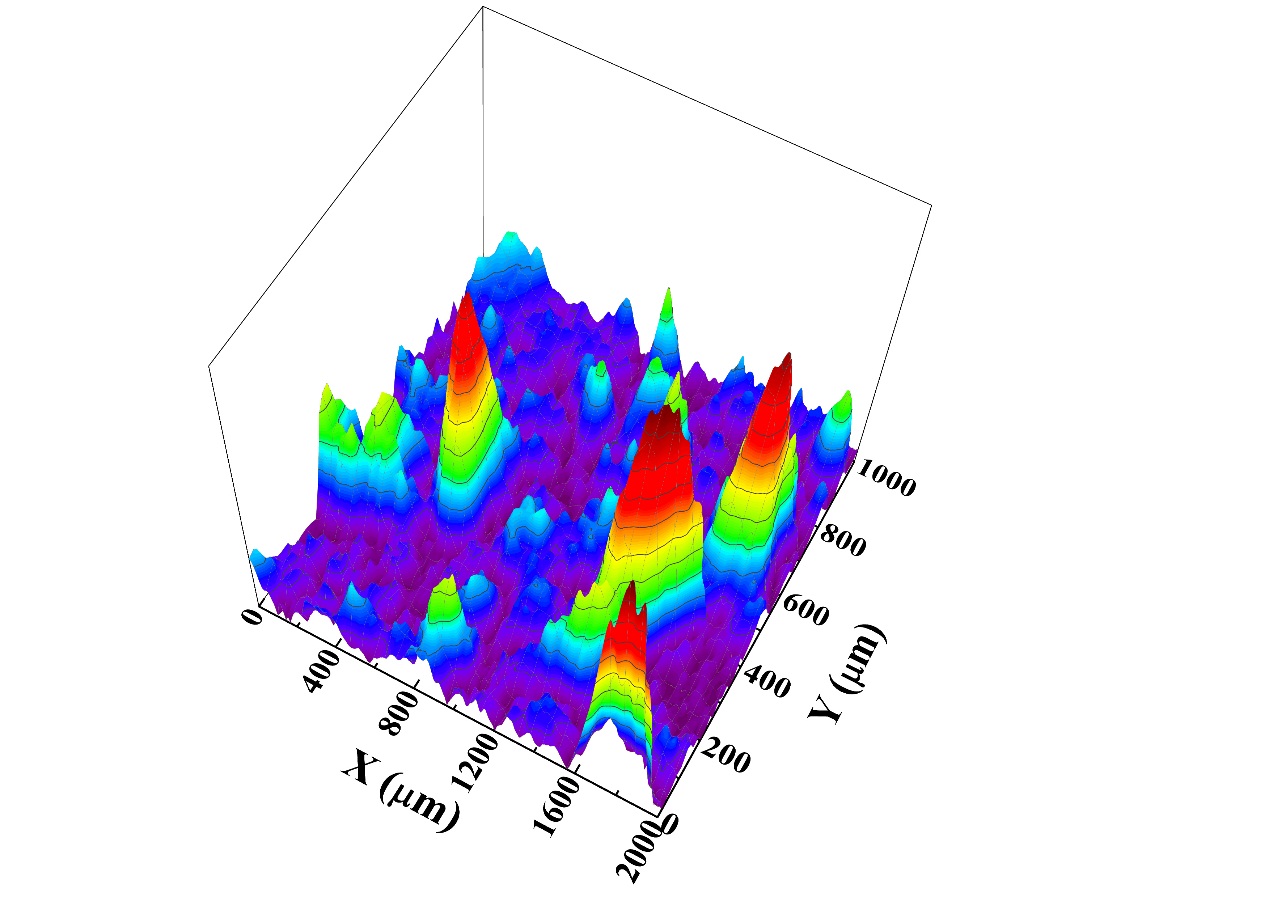

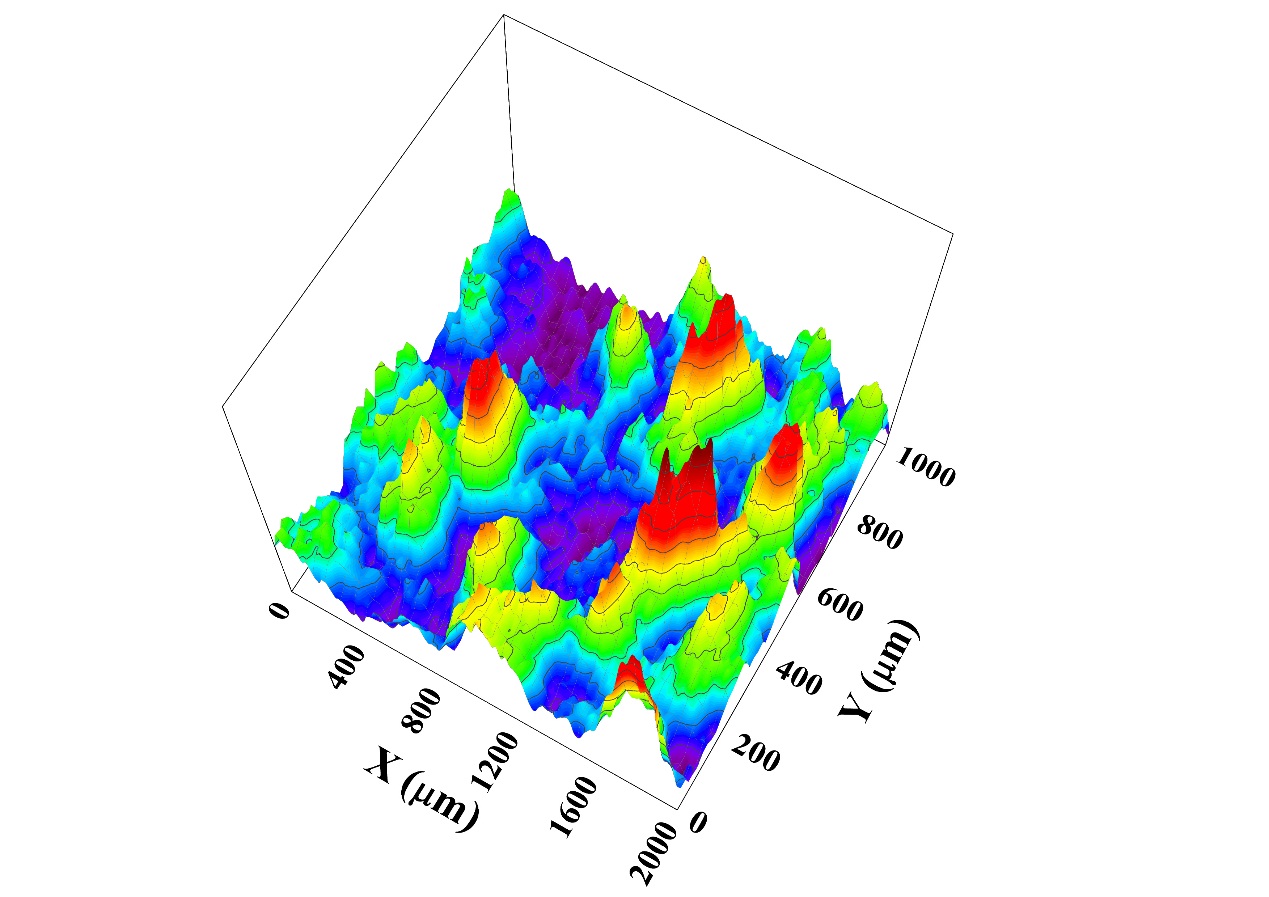
**

**Extended Data Figure 3. Position dependent OIRD signals of sandstone.** Real part intensities at different scanning positions (up), where the Re{*Δp*-*Δs*} values revealed several peaks. Shale’s imaginary part intensities at different scanning positions (down). In the 3-D systems of both the real and imaginary parts, no bedding information can be observed, and instead, the signals indicated overall uniformity. Differently sized sand grains are randomly distributed on the sandstone surface. Large granules can have widths as large as ~ 400 µm. The isotropy properties of the sandstone and some single particles can be clearly observed.


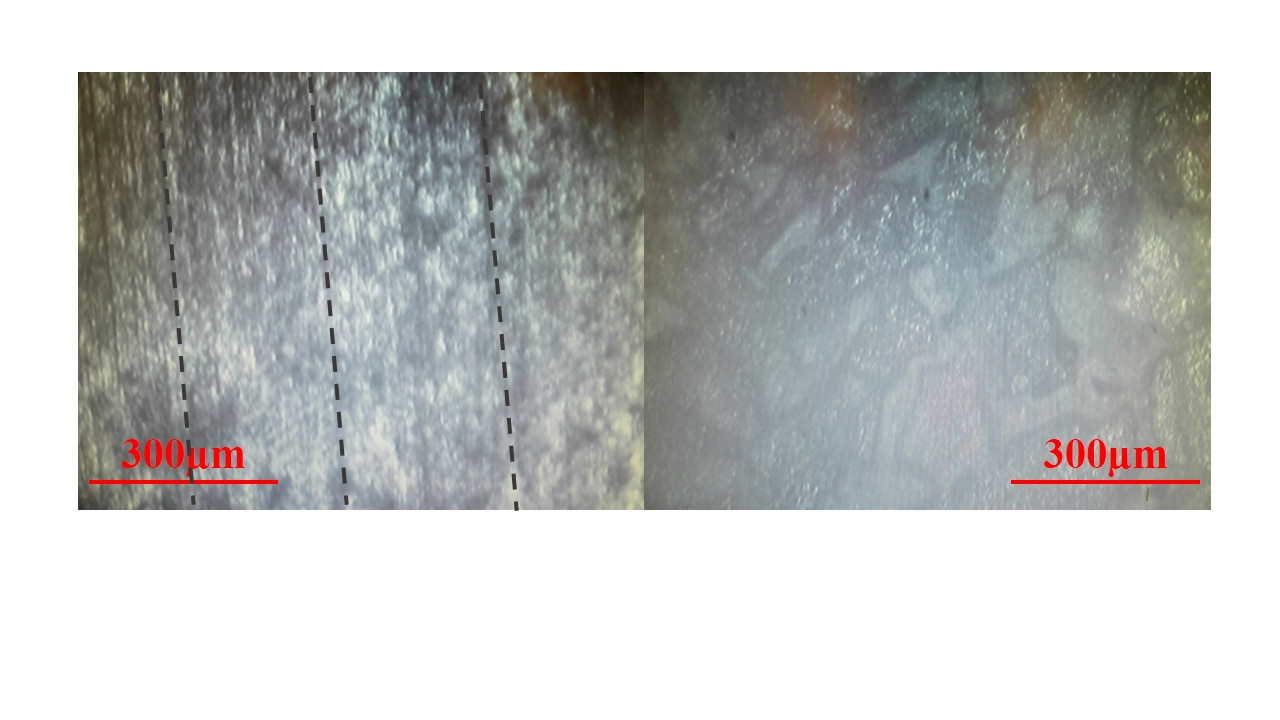


**Extended Data Figure 4. Optical microscope analysis of the surfaces of rocks.** Microscope pictures of the surfaces of shale (left) and sandstone (right). In the shale, the laminations can be clearly observed. The main beddings are depicted with black dotted lines, and the laminations are parallel to each other, with an interval of ~ 300 µm between the adjacent bedding lines. These features are in good agreement with those in Extended Data Figure 2. Meanwhile, among the main laminations, a number of secondary striations are discontinuous and parallel to each other. High homogeneity can be observed between the features. The sandstone surface includes with some random sand grains and can be considered to be isotropic. The OIRD signal was strongly sensitive to the features shown in Fig. 2. Herein, the optical microscope image shows these features and reveals that the sandstone surface is indeed isotropic. The OIRD method thus constitutes a simple and direct approach for the detection of surface properties, including isotropy and anisotropy caused by the chemical components and structures of rocks, such as shale and sandstone.


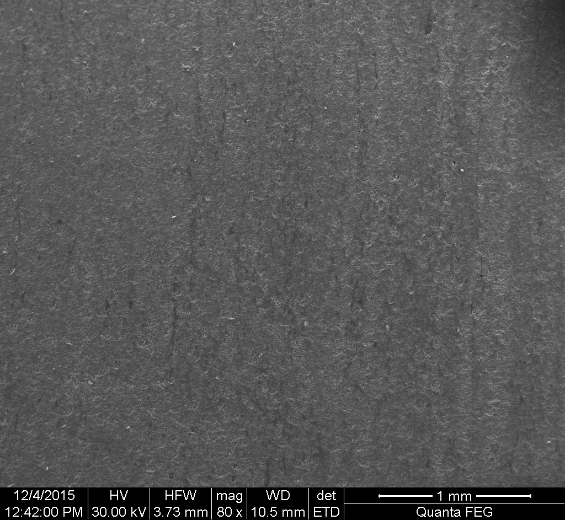

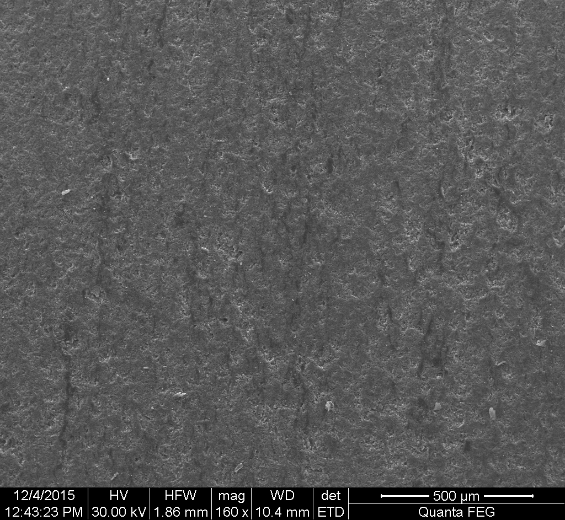

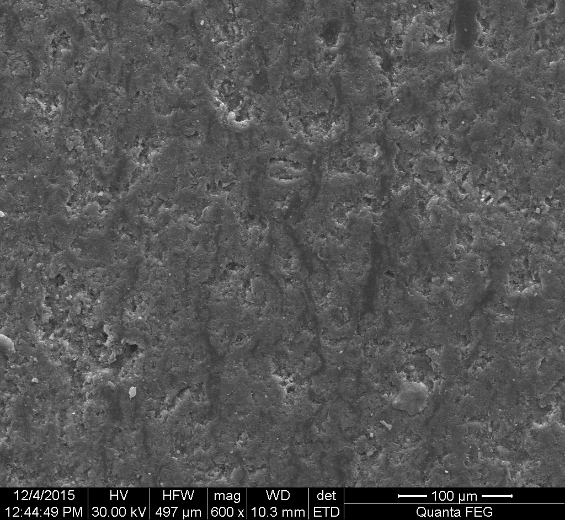

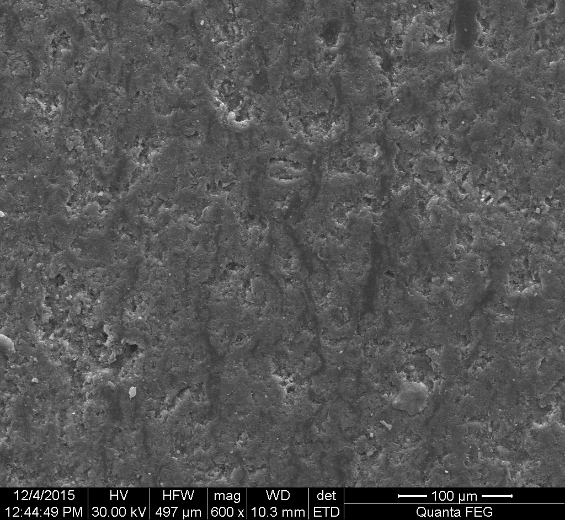

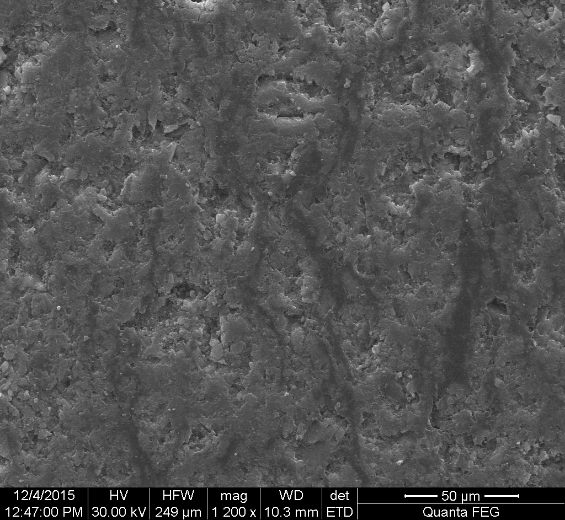

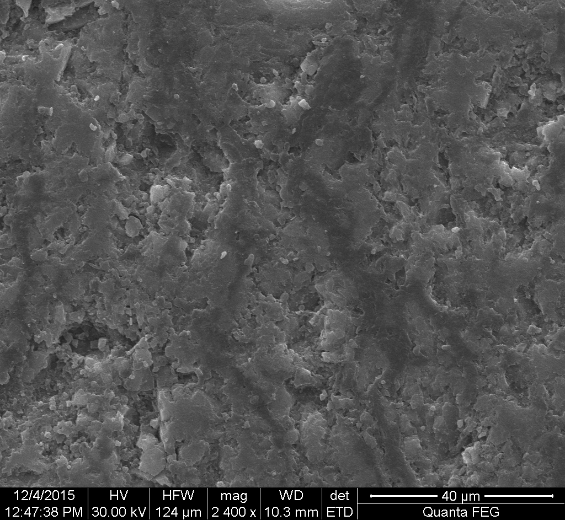

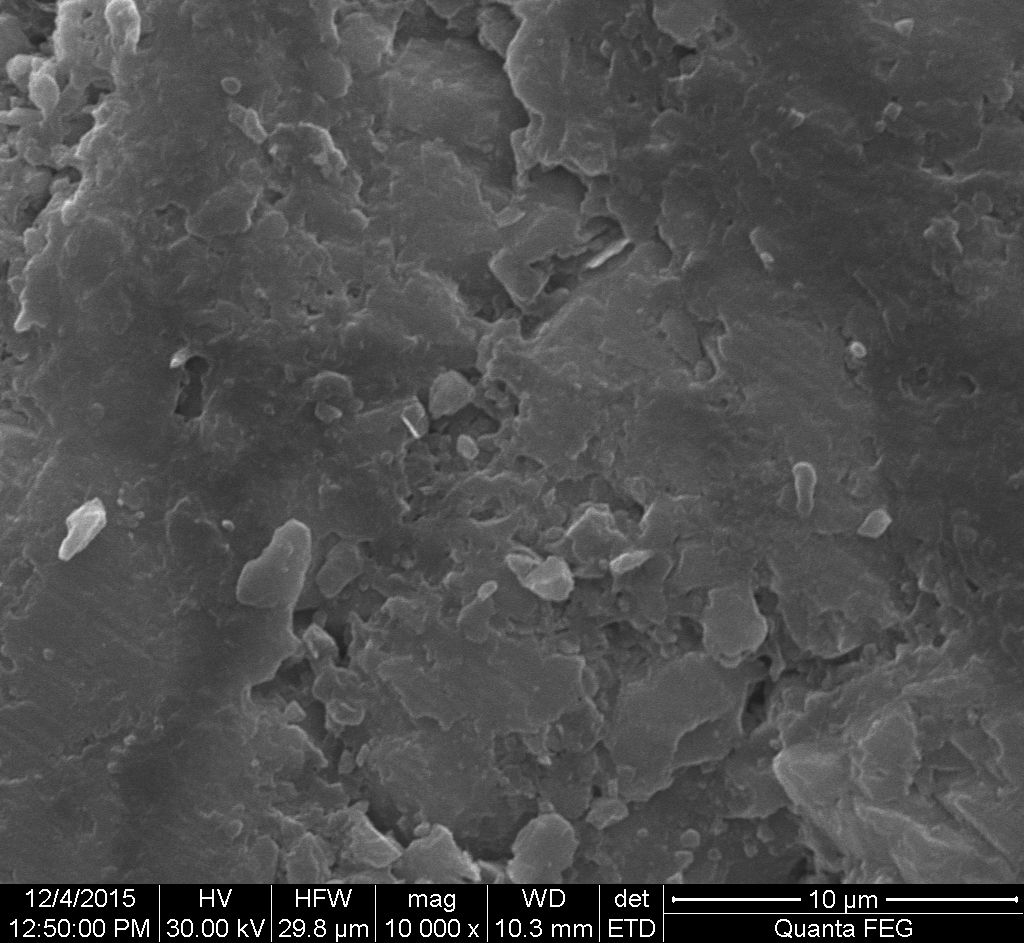

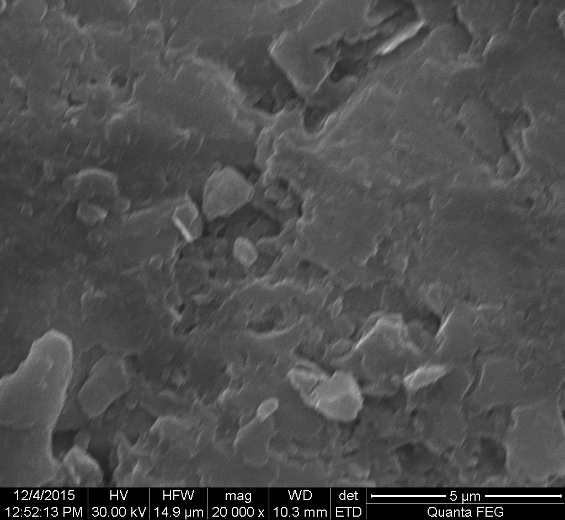


**Extended Data Figure 5. SE imaging analysis of shale.** The first images shows the shale surface on the millimeter scale. A number of parallel stripes can be observed. This area is gradually enlarged, from left to right and from top to bottom. As the amplification factor increases, some micro-cracks with micron widths are gradually observed. In SE imaging, micro-cracks are clearly distinguished in grayscale; in the bottom-right image, the approximately straight crack in the middle of picture is related to that shown by the OIRD peaks in Fig. 1.


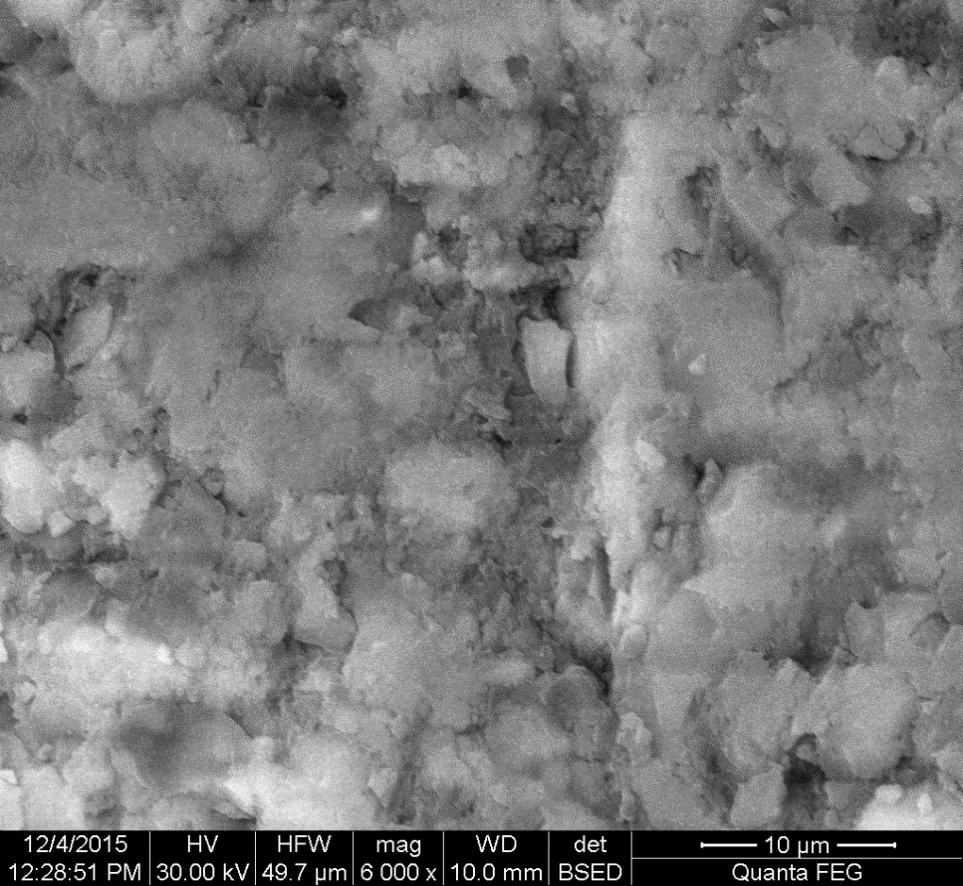


**Extended Data Figure 6. BSE imaging analysis of shale surface.** The white areas validated the orientated arrangement of a material with a relatively large atomic mass that has the same orientation direction as the cracks shown in Extended Data Figure 5.


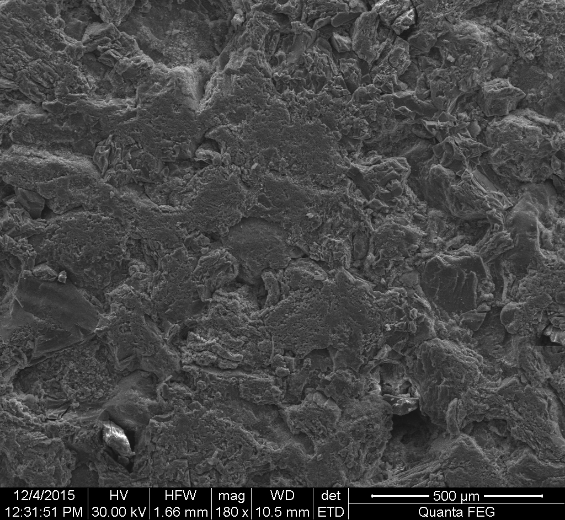

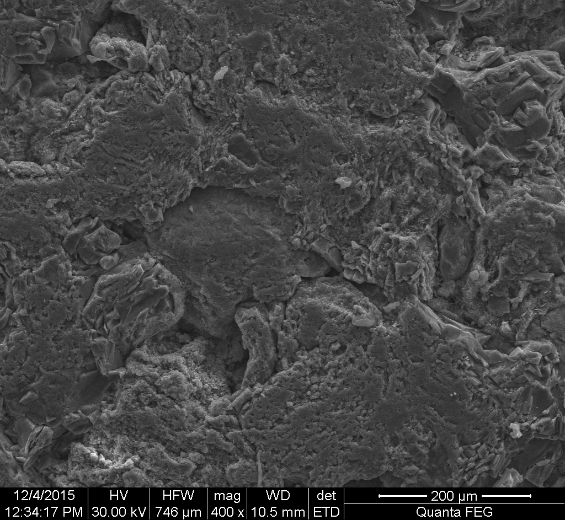

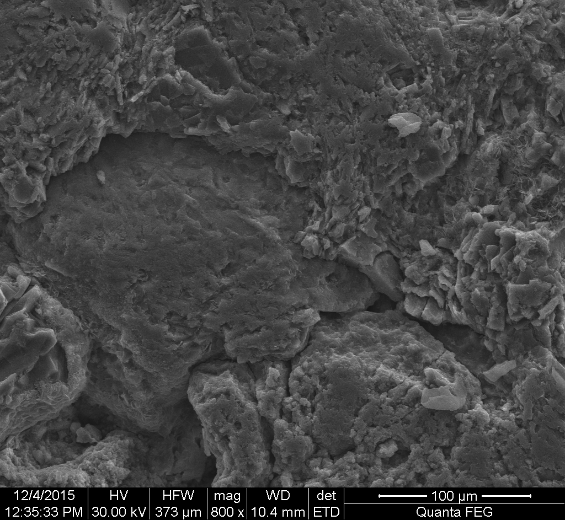

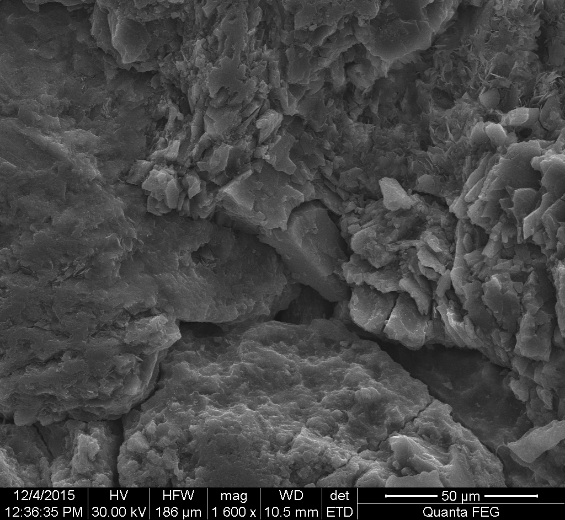

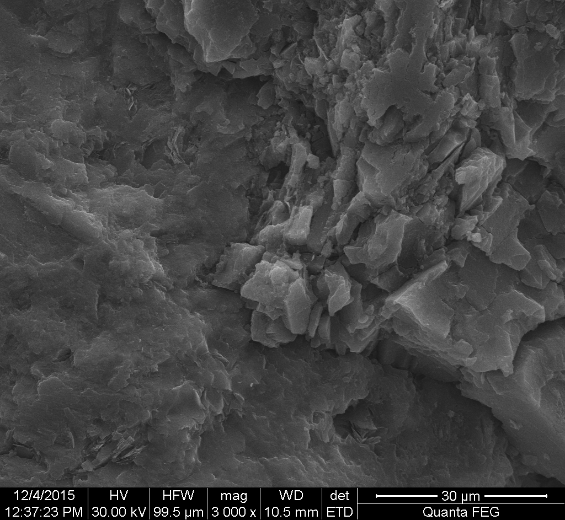

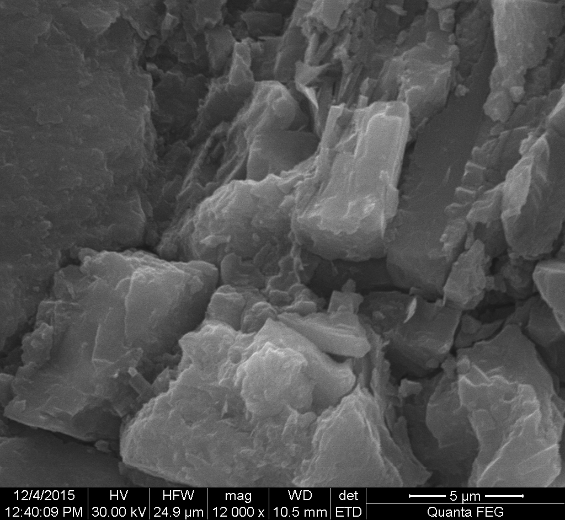


**Extended Data Figure 7. SE imaging analysis of sandstone.** The first image shows the sandstone surface on the millimeter scale. This area is gradually enlarged from left to right and from top to bottom. As the amplification factor increases, some micro-cracks between particles can be gradually observed. The SE imaging shows that differently sized particles are separated by annular cracks. The crack shape in sandstone is different from that in shale. Overall, sandstone can be considered as an isotropic material because of the absence of orientation, in agreement with the information shown in Fig. 2.


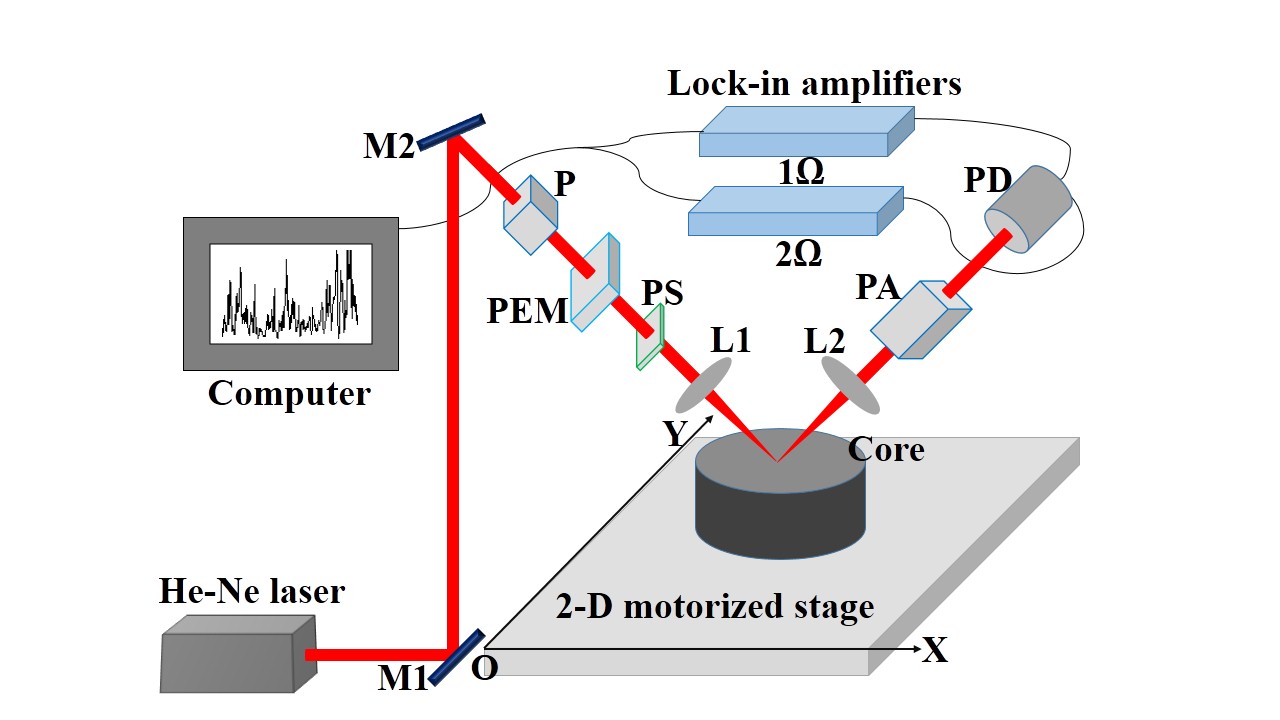


**Extended Data Figure 8. Schematic diagram of OIRD system used to analyze shale and sandstone.** The rocks is fixed on a pair of translation stages that can be moved in the *x* and *y* directions: PEM, photoelastic modulator; PS, Phase shifter; and PD, photodiode. The PEM oscillates the polarization of the laser beam between *p-* and *s-*polarization at a frequency of 50 kHz. The PS adjusts the phase difference *Φ_ps_* between the *p-* and *s-*polarization components. The PD detects the intensity of the reflected laser beam.


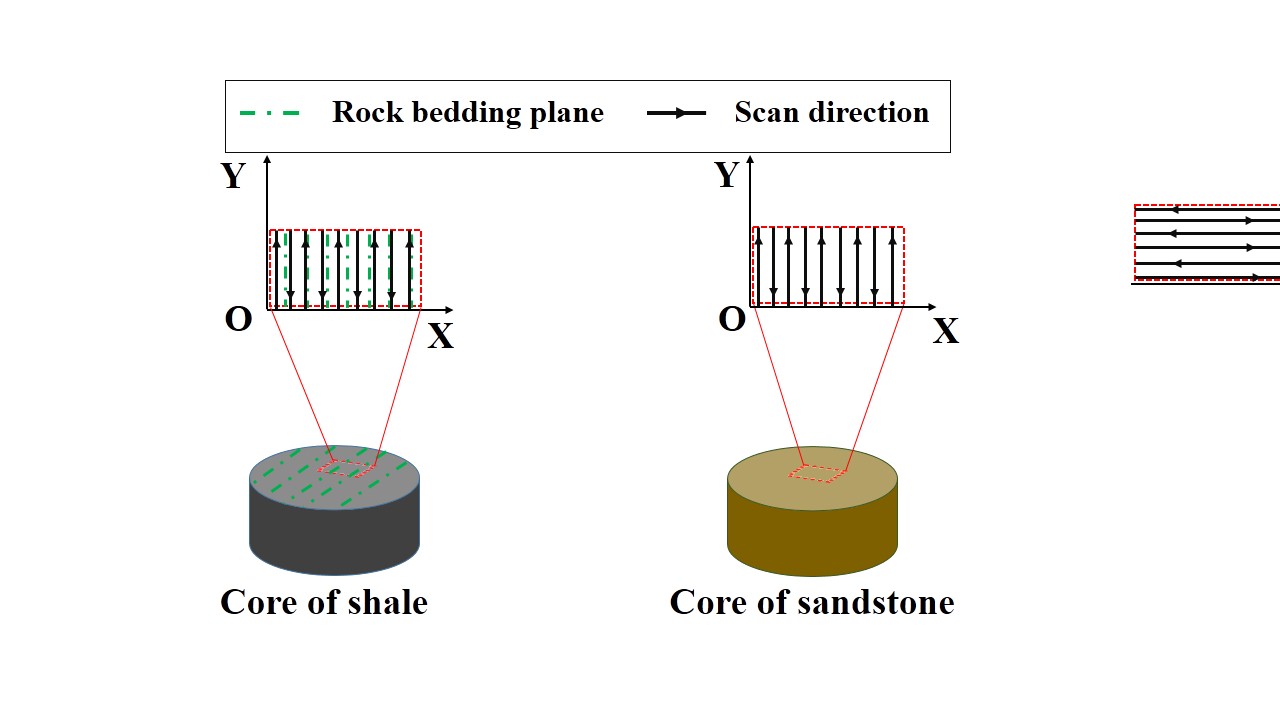


**Extended Data Figure 9. Schematic diagram of the OIRD scan.** Laser scanning of the surfaces of shale (left) and sandstone (right). The rock is fastened to the upper surface of the stage, which is controlled by special software. The interval between two adjacent scans was 4 µm. The scan directions of the laser or the movement directions of the rocks were specifically chosen.
